# Study protocol: a randomised controlled trial of cognitive remediation for a national cohort of forensic mental health patients with schizophrenia or schizoaffective disorder

**DOI:** 10.1186/s12888-016-0707-y

**Published:** 2016-01-13

**Authors:** Ken O’Reilly, Gary Donohoe, Danny O’Sullivan, Ciaran Coyle, Ronan Mullaney, Paul O’Connell, Catherine Maddock, Andrea Nulty, Padraic O’Flynn, Carina O’Connell, Harry G Kennedy

**Affiliations:** Department of Psychiatry, Trinity College Dublin, Dublin, Ireland; National Forensic Mental Health Service, Central Mental Hospital, Dundrum, Dublin 14 Ireland

**Keywords:** Schizophrenia, Forensic mental health, Cognitive remediation, CRT, Neurocognition, Effectiveness, Clinical trial

## Abstract

**Background:**

Evidence is accumulating that cognitive remediation therapy (CRT) is an effective intervention for patients with schizophrenia or schizoaffective disorder. To date there has been no randomised controlled trial (RCT) cohort study of cognitive remediation within a forensic hospital. The goal of this study is to examine the effectiveness of a trial of cognitive remediation for forensic mental health patients with schizophrenia or schizoaffective disorder.

**Methods:**

An estimated sixty patients will be enrolled in the study. Participants will be randomised to one of two conditions: CRT with treatment as usual (TAU), or TAU. CRT will consist of 42 individual sessions and 14 group sessions. The primary outcome measure for this study is change in cognitive functioning using the MATRICS Consensus Cognitive Battery (MCCB). Secondary outcomes include change in social and occupational functioning, disorganised symptoms, negative symptoms, violence, participation in psychosocial treatment and recovery. In addition to these effectiveness measures, we will examine patient satisfaction.

**Discussion:**

Cognitive difficulties experienced by schizophrenia spectrum patients are associated with general functioning, ability to benefit from psychosocial interventions and quality of life. Research into the treatment of cognitive difficulties within a forensic setting is therefore an important priority. The results of the proposed study will help answer the question whether cognitive remediation improves functional outcomes in forensic mental health patients with schizophrenia or schizoaffective disorder. Forensic mental health patients are detained for the dual purpose of receiving treatment and for public protection. There can be conflict between these two roles perhaps causing forensic services to have an increased length of stay compared to general psychiatric admissions. Ultimately a focus on emphasising cognition and general functioning over symptoms may decrease tension between the core responsibilities of forensic mental health services.

**Trial Registration:**

ClinicalTrials.gov Identifier: NCT02360813. Trial registered Feb 4th 2015 and last updated May 1^st^ 2015.

## Background

Forensic Mental Health Services (FMHS) provide treatment for a minority of people with mental illnesses such as schizophrenia who come into contact with law enforcement agencies as a consequence of their mental disorder, or who cannot be safely managed within another service and require specialised therapeutically safe and secure care and treatment for a period of time [1, 2]. The offences carried out by mental health patients are heterogeneous and range from public order offences to homicide. It is possible to divert mentally ill patients charged with less serious offences to general psychiatric services especially when detention in prison would be detrimental to their health [3]. Forensic patients are often judged to have lacked mental capacity to form a criminal intent at the time of the offence. These patients are deemed to be not responsible or diminished in responsibility for what they have done due to deficits in comprehension, reasoning, and judgment [4]. The ‘insanity defence’ available in some jurisdictions is a special example of a loss of capacity within the context of criminal charges such as homicide or serious assault. Patients facing criminal charges and who receive a verdict of not guilty by reason of insanity are admitted to a forensic hospital so that they can receive treatment and to ameliorate the risk of future violence [2]. Frequently the dual role of providing treatment and public protection is codified in law as is the case for the Republic of Ireland’s Criminal Law (Insanity) Act (2006) section 11(2) [5]. In these circumstances independent tribunals tasked with reviewing patients’ detention are asked to consider the welfare and safety of the person and also the public interest (Criminal Law Insanity Act 2010). Forensic mental health services therefore have the dual role of treating and caring for the patient and representing their interests, whilst simultaneously protecting the public from further harm through involuntary detention and risk management [6, 7].

### Length of stay within Forensic Mental Health

Forensic mental health patents are typically hospitalised for longer periods than their non-forensic counterparts [7–12]. International comparisons of length of stay are difficult to establish because they are hampered by differences in patient groups and criminal law [7] For the Republic of Ireland the vast majority of forensic mental health patients have a diagnosis of schizophrenia or schizoaffective disorder with a small minority having bipolar or depressive disorder. A diagnosis of personality disorder would not ordinarily meet the criteria of mental disorder under Irish law and would thus not qualify to receive compulsory mental health care in either the civil or forensic services (Mental Health Act 2001 and Criminal Law (Insanity) Act 2006). Even when acknowledging differences in patient populations it would not be unusual for patients to be detained within a European context for periods greater than five years [12] It is likely that the dual role played by forensic mental health services regarding the needs of the patient on the one hand and society on the other is a contributing factor to lengthy admissions [6].

### Limitations of pharmacotherapy for treating schizophrenia

The primary treatment strategy for patients with schizophrenia or schizoaffective disorder is pharmacotherapy using antipsychotic medication, a proven and efficacious intervention for the positive symptoms of schizophrenia i.e. delusions and hallucinations [13]. Following initial gains however, pharmacotherapy has limited efficacy for improving patient functioning. Antipsychotics are not effective for treating the neurocognitive deficits associated with schizophrenia such as problems with attention, memory and executive functioning; nor do they have efficacy for treating stable, trait-like social cognitive deficits such as emotional perception, theory of mind, context sensitive processing, or emotional reasoning [14]. Antipsychotics have also limited efficacy for treating negative symptoms such as avolition, anhedonia, apathy, blunted affect, asociality, and alogia [15, 16]. It is the neurocognitive impairment, social cognitive impairment, and negative symptoms experienced by patients with schizophrenia that are the strongest contributors to functional outcome [17–20]. Meta-analyses consistently demonstrate that both neurocognitive and social cognitive deficits in addition to negative symptoms account for more of the variance of suboptimal functioning than positive symptoms [17–26]. Specifically neurocognitive and social cognitive difficulties affect the ability to live independently, to engage in meaningful work and to benefit from psychosocial treatment programs. Ultimately these impairments impact on patients’ quality of life [27, 28]. Also negative symptoms are probably partially attributable to cognitive impairments [29]. Because of the centrality of neurocognitive problems for patient functioning and because neurocognitive and social cognitive deficits occur prior to the onset of psychotic symptoms it has been argued that schizophrenia should be reconceptualised as a cognitive rather than a psychotic disorder [27]. Moreover it has been suggested that the development of new therapies for improving functional outcomes for patients with schizophrenia has been impeded by emphasising the psychotic features of the disorder [27].

### Limitations of psychological and occupational interventions within forensic mental health

To address patients’ suboptimal functioning and violence risk factors, forensic mental health services use an eclectic mix of occupational therapy and psychosocial treatment programmes. Many of these interventions have a limited evidence base within forensic mental health practice [30–35]. But some specific programmes such as ‘reasoning and rehabilitation’ have been formally assessed, using violent behaviour and attitudes as outcome measures [36, 37]. Also concern has been expressed that patients with schizophrenia and other psychotic disorders may not be able to benefit from such programmes due to their negative symptoms and cognitive deficits [19, 28]. Recently we found that a nationally representative cohort of forensic mental health patients with schizophrenia and schizoaffective disorder scored more than three standard deviations below the population mean on the MATRICS Consensus Battery for cognitive deficits in schizophrenia [38]. Amongst patients with schizophrenia difficulties can occur at any point of the informational processing stream [39, 40]. Therefore forensic mental health patients with schizophrenia may not possess the necessary motivation and basic cognitive abilities to attend to the information being presented, store the information within their memory and utilise the information when presented with future problems and challenges. But because psychological interventions have a robust evidence base for a variety of mental disorders [41], it is probable that patients with schizophrenia could benefit from such interventions if their cognitive abilities could be partially remediated, or if their self-efficacy and intrinsic motivation were enhanced.

### Cognitive remediation therapy

One approach that has shown potential to improve patients’ cognitive and motivational difficulties in non-forensic settings is cognitive remediation therapy [42–44]. Cognitive remediation therapy is a behaviourally based training approach designed to help patients improve their cognitive abilities and real world functioning. A variety of therapies exist under the cognitive remediation umbrella but most aim to either strengthen patients basic cognitive capacities through a process of drill and practice, or to teach patients more effective ways to deploy cognitive resources using meta-cognitive strategies. Cognitive remediation is a nonthreatening activity which patients enjoy and focuses on success and mastery experiences and therefore has the potential to increase self-efficacy [45]. A recent meta-analysis by Wykes has demonstrated that cognitive remediation is an effective intervention for patients with schizophrenia [42]. Within the Wykes meta-analysis, the average patient with schizophrenia or schizoaffective disorder who received cognitive remediation improved performance on cognitive tasks by an effect size of about .5 (Cohens d) and .42 on patient functioning. Also cognitive remediation therapy has been shown to produce durable improvements in cognition and functioning [42]. And there is evidence that cognitive remediation can optimise patients’ responses to psychosocial rehabilitation [46].

But the evidence base for cognitive remediation within a forensic mental health setting is limited. To date only two randomised trials have been conducted. One study investigated the feasibility of improving social cognition amongst forensic mental health patients [47]. The second study mixed forensic mental health patients with general mental health patients. Mixing general mental health patients with forensic patients may undermine the confidence with which the findings can be generalised to forensic mental health patients as a whole [48]. Of note within this study the forensic mental health patients were significantly more cognitively impaired on working memory and verbal learning than the general mental health patients. However both studies produced positive outcomes on a range of measures including recognising emotion, neurocognition, aspects of patient functioning and patient satisfaction. Cognitive remediation therefore may be a promising intervention for forensic mental health patients. In theory cognitive remediation approaches have the potential not only to improve patients’ cognitive abilities and day to day functioning, but also contribute to patients’ ability to benefit from additional psychosocial and violence risk reduction programmes, thereby enhancing recovery and perhaps reducing length of stay.

### Functional capacity and public protection

The separation between patient care and treatment, and public protection may be a false dichotomy. Although the link between violence and schizophrenia is typically attributed to psychotic symptoms such as delusions and hallucinations, many violence risk factors within this population concern suboptimal functioning [49–51]. Violence risk prediction or violence proneness schemes take advantage of this and place the same weight on items concerning suboptimal functioning as on items concerning psychotic symptoms or other risk factors [51]. Homelessness, employment problems, relationship difficulties, substance misuse, stress etc. are all risk factors for violence [50]. Many of the violence risk factors associated with suboptimal functioning are probably related to the cognitive difficulties experienced by patients with schizophrenia. It may also be that forensic patients are more functionally impaired than their non-forensic counterparts thus increasing their violence proneness and creating the circumstances for them to come into contact with law enforcement agencies [38]. Using a prospective cohort design, we have recently demonstrated that deficits in neurocognition and social cognition accounted for a large portion of the variance of reactive violence carried out by forensic hospital patients with schizophrenia [38]. We also found that neurocognitive deficits act as a distal risk factor whose effects on reactive violence were mediated by more proximal factors such as problems with social reasoning, impaired functioning, symptoms and violence proneness [38].

### Prioritising patient functioning over symptoms

Improving and where possible restoring patient functioning is central to psychiatric care. But services may be prone to emphasising the medical treatment of symptoms over interventions designed to restore patient functioning. For instance, a recent investigation by the Schizophrenia Commission into the provision of care for people with psychosis in England found inpatient settings to be “anti-therapeutic” with medication being prioritised over psychological interventions [52]. Concerning community care and treatment, one retrospective longitudinal study examining 25,000 Swedish patients with schizophrenia found an increase in the rate of adverse outcomes from 1972 to 2009. Amongst this cohort there was an increase in premature death, violent crime, and suicide [53]. The increase in the number of adverse outcomes was notably associated with a decrease in the numbers of inpatient beds and an increase of patients living in the community [53]. This finding may be explained by pressure to discharge patients who do not possess the necessary functional capacity to cope in the community, but have received antipsychotic medication. Clearly patients with low levels of functioning need high levels of support delivered in either an appropriately resourced community or hospital setting. But by placing greater emphasis on treating and managing the cognitive difficulties that underpin functional impairments, it may also be possible to reduce violence and other adverse outcomes. Although forensic mental health services have a dual responsibility to provide care, in addition to managing and decreasing violence risk, these are not necessarily conflicting roles. The prioritisation of cognition and function over symptoms may resolve the conflict between treatment and public protection. Any conflict that does occur between care and public protection is more likely to be a by-product of the limitations of particular treatment approaches and conceptual paradigms. Because a focus on the cognitive and functional deficits experienced by forensic mental health patients has the potential to bring the twin goals of patient care and public protection into alignment, evaluating the effectiveness of cognitive remediation is an important research priority.

### Current study

To date there has been no randomised controlled cohort study evaluating cognitive remediation therapy within a forensic hospital although there have been feasibility and mixed studies [47, 48]. Therefore an important objective is to conduct a trial of cognitive remediation therapy examining the effectiveness, functional outcomes and patient satisfaction for a nationally representative cohort of forensic mental health patients. We do not know whether cognitive remediation is an effective rehabilitation approach within this setting for improving cognition, reducing negative symptoms and improving general functioning. Equally it is not clear whether cognitive remediation has the ability to synergistically combine with routine psychosocial and violence risk management programs and to enhance patients’ ability to benefit from these interventions. Finally, it is not clear whether forensic mental health patients would find it acceptable to participate in an intensive programme of cognitive remediation.

### Hypotheses

The current study will aim 1) to test the efficacy of cognitive remediation therapy for improving patient cognition, symptoms and functioning, where functioning includes measures of participation in psychosocial treatment programmes, recovery, and dynamic violence risk; 2) to establish patient satisfaction with cognitive remediation therapy within a forensic setting.

## Method

The method was informed by the Clinical Trials Assessment Measure for psychological treatments, which is an instrument designed to assess the quality of psychological trials [54]. The Clinical Trials Assessment Measure covers the domains of sample characteristics; allocation to treatment (including allocation concealment, blinding, and randomisation); comparison treatments; outcome assessment (including standardised outcomes and blinding of participants); treatment description (including protocol and fidelity assessment); and appropriate analysis (such as intention-to-treat analysis). The validity measures for adherence to the protocol will include rate of enrolment, rate of retention, tests of the success of blinding, and the number of patients who complete the primary outcome measures.

This is a single centre randomised controlled trial.

### Ethics, consent and permissions

The study was approved by the Research, Ethics and Audit Committee of the National Forensic Mental Health Service (NFMHS) and the School of Medicine Ethics Committee, Trinity College Dublin. All patients participating in the study will provide informed signed consent.

### Setting

The NFMHS provides specialised care for adults who have a mental disorder and are at risk of harming themselves or others. At the time of the study the NFMHS had 94 secure inpatient beds located on a single campus (The Central Mental Hospital, CMH), and 13 community beds. The CMH is the only secure forensic psychiatric hospital for the Republic of Ireland, a population of 4.6 million.

### Participants

Approximately sixty patients under the care of the Central Mental Hospital will be recruited to participate in this study. Inclusion criteria are having a diagnosis of schizophrenia or schizoaffective disorder established using the Structured Clinical Interview for the Diagnostic and Statistical Manual IV (SCID) [55] and being proficient in English. Exclusion criteria are being acutely psychotic, being judged too dangerous to participate in treatment (positive symptoms combined with aggressive or self-harming behaviour in the last month) or being over 65 years of age. Inclusion criteria are broad and exclusion criteria are minimal because we are primarily interested in investigating whether CRT will be effective for a nationally representative cohort of forensic mental health patients.

### Randomisation and treatment allocation

**Fig. 1:**
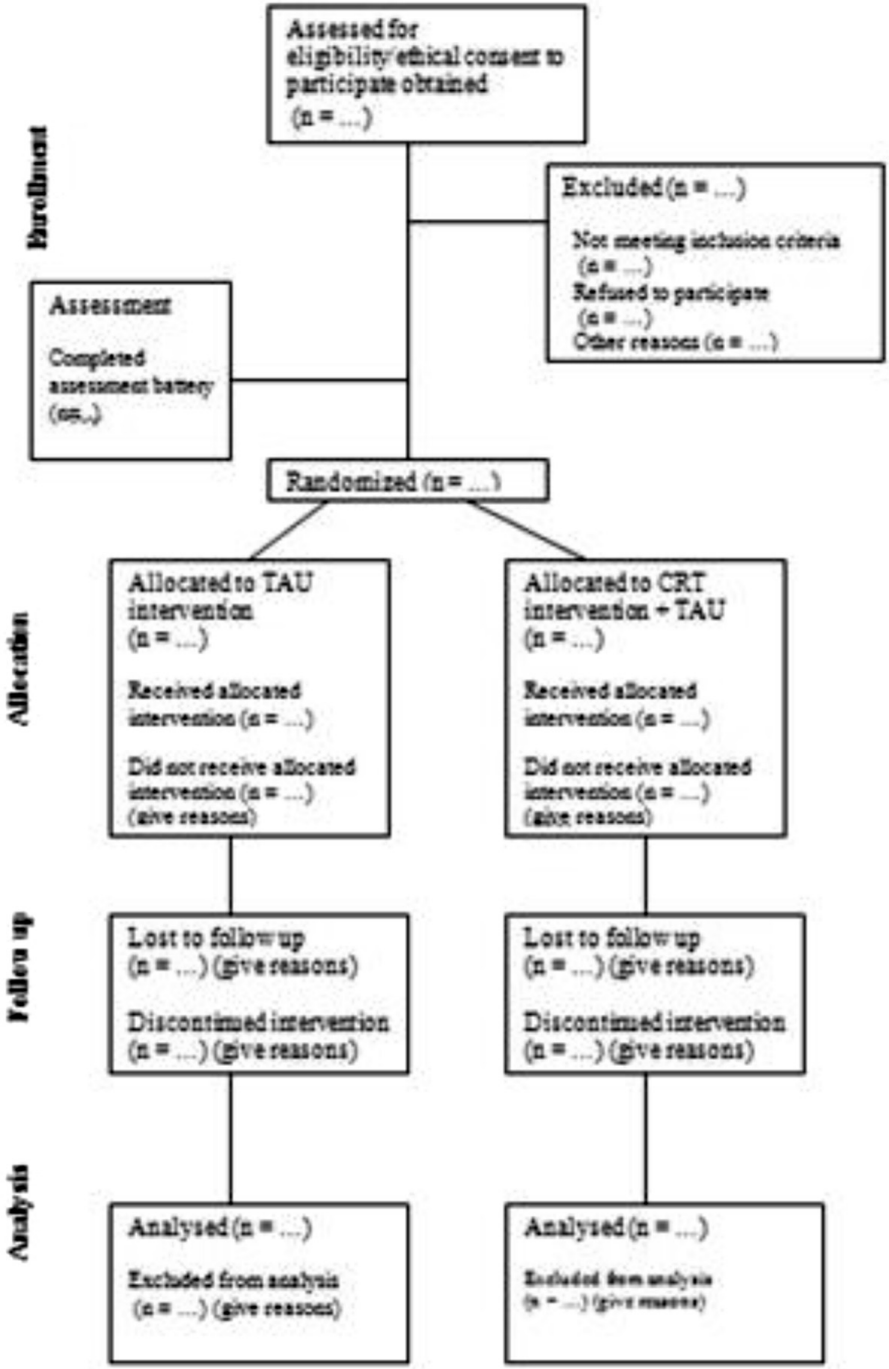
Study outline. Following obtaining of ethical consent, participants were assessed and then randomised into the treatment as usual (TAU) or cognitive remediation (CRT) and TAU interventions

Upon enrolment in the study participants will be randomised to cognitive remediation and a waiting list control group receiving treatment as usual (TAU) by the clinical director of the Central Mental Hospital using ‘select cases’ ‘random samples’ (select cases random number generator) function in SPSS V21 [56]. Fig. 1 is a CONSORT diagram outlining patient allocation. Patients randomised to receiving TAU will be offered the intervention upon completion of the study. The clinicians conducting the therapy sessions will be different from the research team carrying out the assessments. The sequence of randomisation will be concealed from the research team carrying out the baseline and outcome assessments i.e. blinded assessment. All participants will be trained not to reveal their study condition prior to each follow up assessment. Should the blind be broken this will be noted. Furthermore all assessors will be asked to guess whether participants were receiving TAU or CRT for each of the follow up assessments to see if they perform at a chance level of accuracy. Patient participation in CRT will be shared with their treating psychiatrist. The socio-demographic characteristics of the cognitive remediation group and the waiting list control group will then be compared on a range of measures e.g. age, sex, dose of antipsychotic medication (expressed as chlorpromazine equivalents) [57–59], pre-morbid IQ (estimated from TOPF^uk^) [60], MATRICS global composite scores, MATRICS domain scores, Social Cognitive Scores [60–62], real world functioning (SOFAS), psychiatric symptoms (PANSS), negative symptoms (CAINS) and violence proneness (HCR-20) [63–66].

### Cognitive remediation therapy

Cognitive remediation therapy is a behaviourally based training programme designed to improve cognitive problems associated with schizophrenia and schizoaffective disorder [42–44]. Patients allocated to cognitive remediation will receive three individual sessions a week and one group session for approximately 14 weeks, 56 sessions in total.

The focus of the individual sessions is to enhance patients’ basic cognitive abilities through drill and practice and to introduce patients to a variety of meta-cognitive strategies to compensate for reduced performance according to need [44]. Meta-cognitive strategies may be distinct for particular cognitive domains e.g. the strategies of problem identification, breaking problems into parts, brainstorming, sequencing, monitoring, reflecting may be particularly useful for problem solving. Whereas strategies such as visualisation, chunking, association, rehearsal etc. may be helpful to compensate for memory difficulties. And strategies like self-verbalisation may be useful to enhance attentional abilities.

The focus of the group sessions is to help patients normalise and develop insight into their cognitive difficulties, to receive support and encouragement, and to generalise gains [67]. A detailed manual has been developed to guide the delivery of the CRT support group.

### Cognitive remediation operationalised by nine treatment principles

Our cognitive remediation therapy is a principle driven intervention consisting of nine treatment principles (Table 1) and is in keeping with the recommendations of a task force on Principles of Therapeutic Change that Work, sponsored by the American Psychological Association and the North American Society for Psychotherapy Research [68]. In addition to the CRT literature the approach is also influenced by the success of multi-systemic therapy (MST), an empirically supported treatment for conduct disorder. Multi-systemic therapy is a flexible intervention operationalised by treatment principles rather than a prescriptive session format [69]. Conduct disorder, like schizophrenia is a heterogeneous disorder and also has a history of being considered refractory to psychological interventions. In contrast to manualised protocols, principle driven interventions like MST aim to provide clinicians with flexible heuristics rather than a set of tightly defined procedures prescribed at specific times in therapy. The emphasis on broad treatment principles rather than a scripted session format is therefore to facilitate a patient centred approach, which is responsive to each patient’s own unique strengths and vulnerabilities.

**Table 1:** Principles guiding cognitive remediation intervention

**Principle 1, Relationship Building:** A major focus of each session is to prioritise the development of a strong therapeutic relationship. The therapeutic relationship will be strengthened by providing a credible rational for participation, explicitly linking the cognitive remediation to patients’ goals, promoting success experiences, making participation enjoyable, providing positive reinforcement, and managing ruptures, which may occur during the course of the intervention. **Principle 2, Collaborative Goal Setting:** So as to promote ‘buy in’ patients will be encouraged to develop a series of short term, medium term, and long term goals. Patients neuropsychological and risk assessments e.g. HCR-20, Dundrum toolkit, will be shared with patients to create a platform to develop goals. An explicit connection will also be drawn between cognitive difficulties and patients’ aspirations. Short term goals may include having the concentration required to watch a TV programme or to read a book. Medium term goals may include patients’ ability to self-medicate or move to a less secure unit. Long term goals may include returning to work, and developing relationships outside the hospital. **Principle 3, Session Structure:** Each session will begin with a mood check to establish rapport or identify problems followed by agenda setting, implementation of the agenda items, and summaries before moving on to the next agenda item. The session will end by giving patients the opportunity to provide feedback. **Principle 4, Content of the sessions:** The sequencing of interventions will be informed both by patients goals and their unique strengths and vulnerabilities as documented by neuropsychological assessment. Cognitive domains at the start of the informational processing stream e.g. attention and vigilance, working memory etc will typically be prioritised over those occurring later e.g. comprehension and social problem solving. This is because difficulties associated with higher level cognitive processes may be a result of problems with more basic processes such as attention and memory. As patients demonstrate some improvement in core cognitive skills, higher level domains will be targeted. Clinical judgement will be required to determine if patients achieve a basic level of mastery in certain cognitive domains of if a ceiling has been reached before progressing to more complex domains. CRT therapists should carefully assess whether patients are improving on core domains e.g. verbal memory etc., and if these improvements are being maintained over time. **Principle 5, Pacing:** Therapists are encouraged to avoid trying to squeeze too much into each session or to work on too many problems simultaneously because it takes time to consolidate skills. In other words, patients need opportunities to repeat tasks again and again to improve performance, which is referred to as massed practice. Throughout the intervention each session should build on the next and be targeted at concrete goals. Patients should be provided with feedback on their progress towards goals. Newly acquired skills should not be abandoned once developed but refreshed during future sessions. Patients may also need breaks between tasks. This down time is a good opportunity to ask patients about their lives and to strengthen the therapeutic relationship. **Principle 6, Errorless Learning and Scaffolding:** Task difficulty should be set so that patients obtain a high level of success on each task to avoid faulty learning and to enhance morale. Patients will be required to obtain a success rate of 80 % before the cognitive demands of the task are increased. Where problems are encountered therapists should provide scaffolding and model successful completion of tasks. **Principle 7, Meta cognitive Strategies:** A major focus of each session will be to explicitly teach patients meta-cognitive strategies which are somewhat independent of basic cognitive ability and can be flexibly applied across situations. Examples of meta-cognitive strategies include goal setting, visualisation, focusing on one thing at a time, self-verbalisation, planning, breaking problems into parts, sequencing, chunking, advantage disadvantage analysis, perspective taking, monitoring performance, reflecting on performance etc. It is particularly important to explicitly model the effective use of meta cognitive strategies for patients. The effectiveness of strategies should be carefully assessed using a behavioural experiment framework. The use of particular strategies should be consolidated as evidenced by generalisation before additional meta-cognitive strategies are introduced. When mastery of basic strategies has been consolidated patients can be encouraged to simultaneously use multiple strategies. **Principle 8, Generalisation:** Patients will be encouraged to utilise their cognitive skills outside of remediation sessions by participating in a support group. The focus of the support group will be helping patients to develop a shared understanding of the cognitive deficits associated with schizophrenia, to develop an awareness of how these deficits affect their lives, to identify situations where they can apply their cognitive skills, to obtain encouragement and support from other members of the group on how to implement these skills, to strengthen narratives where success has been achieved. In addition to the above positive group participation in and of itself may enhance cognitive processes as it requires patients to monitor their thoughts, reframe from interruptions, structure their contributions, and reflect on feedback. **Principle 9, Managing Ambivalence:** Patients ambivalence towards participating should be met in a non-defensive empathic manner. Advantages and disadvantages of participating should be listed using pen and paper to ease the burden on working memory and to model effective problem solving. Patents should be gently reminded of their goals and their initial commitment to participate for the duration of the intervention. Ways of making the cognitive remediation more relevant or enjoyable should be actively explored.	

Our cognitive remediation programme also aims to align the goals of forensic mental health services with the goals of individual patients. For example, forensic mental health services may have a number of goals for their patients concerning physical health, mental health, substance misuse, harmful behaviour and occupational and recreational functioning [70]. These goals may vary in their level of explicitness, consist in a number of sub-goals, and vary in the extent to which they are communicated to patients. Also in some cases the patients may not share the service's goals but rather be a passive participant in the rehabilitative process. For instance, they may not agree that they have a substance misuse problem, have a mental illness or be at a high risk of violence. In these cases, the service, psychiatrist, key-worker, multidisciplinary team and other parties are the customers of the intervention and not the patient. In contrast the starting point of our cognitive remediation intervention is to help patients to clearly and explicitly articulate their goals. Explicit links are then drawn between cognitive difficulties and patient aspirations and cognitive remediation is then offered as a vehicle which can help actualise goals. Every attempt is made to facilitate the patient to take on the role of the customer. We agree with Lindqvist and Skipworth who argue that it is the hopes of the patient which are decisive for recovery [33, 34].

Like MST our nine treatment principles are general statements that can be easily remembered and applied, which identify relational conditions, therapist behaviours, and classes of interventions likely to lead to change. The treatment principles attempt to integrate the specific theories and techniques advocated by the CRT literature e.g. self-verbalisation or monitoring, errorless learning, scaffolding etc. combined with research into what makes psychological interventions effective in general e.g. emphasising the therapeutic relationship, offering a credible rational for treatment, and routinely evaluating progress [41, 71].

Wykes defines self-monitoring, errorless learning and scaffolding as follows [43]. Self-monitoring is a technique for rehearsal of both the task instructions as well as task completion and can be accomplished by using verbalisation either overtly or covertly. Errorless learning is a technique whereby the therapist minimises opportunities for the participant to make errors. For example, individuals only attempt tasks where they have an 80 % success rate. Finally scaffolding is a technique whereby the therapist challenges the participant to complete difficult tasks but with the assistance and guidance of the therapist. Our nine treatment principles are also in keeping with recent developments within the CRT literature, where it is has been found that the therapeutic relationship, emotional state, and the motivation of participants, in addition to an emphasis on skills transfer, all play an important role in treatment success [42, 72–75]. For example, working alliance contributes to the success of CRT [72]; positive mood facilitates creative problem solving [73]; intrinsic motivation can be enhanced by providing a personalised context that links treatment with everyday life, and also by tailoring the intervention to the learning goals of each participant [74, 75]; and functioning outcomes are best achieved by combining CRT with other rehabilitation programmes [44].

It is also hoped that the nine treatment principles will form a bridge between abstract theoretical models and the concrete interventions carried out during sessions. Our approach combines models of cognitive remediation such as drill and practice aiming to strengthen cognitive performance as well as teaching meta-cognitive strategies aiming to compensate for cognitive function, whilst at the same time emphasising the process of therapy e.g. relationship building, goal setting, managing ambivalence etc.

In practical terms each session will involve practicing discreet cognitive functions identified by the MATRICS consensus cognitive battery [63] e.g. attention, working memory, verbal memory, visual memory, comprehension, problem solving and social cognition. A variety of pen and paper materials will be used to achieve this aim. A free open source version of the Dual N-back computer programme (http://brainworkshop.sourceforge.net) will also be used to help patients develop and modulate their attentional recourses in addition to their working memory (visual and spatial) and processing speed. Over the course of the intervention as patients make progress the cognitive remediation procedures will gradually increase in difficulty.

The cognitive remediation will be delivered by Masters’ level assistant psychologists and the cognitive remediation support group will be delivered by multidisciplinary professionals including psychiatric registrars, occupational therapists and psychiatric nurses. All therapists contributing to the cognitive remediation programme will attend a three day training course prior to delivering the intervention. All therapists will attend weekly supervision sessions where fidelity to the treatment principles will be actively monitored. Fidelity to the treatment will also be assessed by observing adherence to the nine treatment principles during randomly selected individual and group treatment sessions.

### Treatment as usual

Participants in both conditions will receive treatment as usual from hospital clinicians. At a minimum, this will consist of antipsychotic pharmacotherapy and a therapeutically safe and secure environment appropriate to the individual patient’s needs [76–78] however most patients are expected to be involved in a range of therapies provided by multidisciplinary team members, including psychiatrists, clinical psychologists, psychiatric nurses, occupational therapists and social workers [78].

Medication will be managed separately by the consultant psychiatrists responsible for the patients’ care and may change over the duration of the study as required. Both antipsychotic dose and anticholinergic burden will be measured at each assessment point as these may be important treatment moderators [79].

The number of routine therapeutic hours each patient receives in the treatment as usual (control) and the cognitive remediation group will be recorded from patient’s progress notes/ medical charts each week. A narrow definition of therapeutic activity will be applied to prevent over inclusion: a therapy will be defined as any activity that is occurring on a consistent or regular basis targeting specific goals and designed to address patients’ forensic mental health needs. From this perspective regular occupational therapy, cognitive behavioural work, psycho-education, harmful behaviour programmes, substance misuse interventions, group programmes etc. would be defined as therapeutic activities, in contrast multidisciplinary team meetings, general interviews or assessments, physical exercise and general vocational or educational work will not be seen as therapies.

### Assessment battery

All assessors will complete a training programme prior to administering study related assessments. For assessments that require clinical judgment (e.g. symptom severity measures), assessors will observe a number of interviews carried out by an experienced consultant psychiatrist whilst simultaneously rating patients' performance. The inter-rater reliability of assessors will also be measured as part of the training programme for assessors. Primary and secondary outcome measures are presented in Table 2.

**Table 2:** Outcome Measures

Primary Outcome Measure	MATRICS Consensus Cognitive Battery Composite Score (MCCB)
Secondary Outcome Measure	MCCB Domain Scores
	Social cognitive assessments
	• The Reading the Mind in the Eyes Test
	• The Faux Pas Recognition Test
	• The MSCEIT
	Social and Occupational Functioning Assessment Scale (SOFAS)
	Positive and Negative Syndrome Scale (PANSS) – negative and disorganised scales
	Clinical assessment interview for negative symptoms (CAINS)
	Historical Clinical Risk -20 (HCR-20)
	DUNDRUM-3 Programme Completion Scale
	DUNDRUM-4 Recovery Scale
Validity Checks	Rate of enrolment, rate of retention, rate of completion of primary outcome measures, success of blinding.
Patient satisfaction survey	Service user developed interview.

### Primary outcome measure: change in global cognitive functioning

Cognitive functioning among study participants will be assessed at baseline, end of treatment (approximately 6 months) and 8 month follow up using the MATRICS Consensus Cognitive Battery (MCCB) global composite score [61]. The MATRICS battery covers seven cognitive domains: processing speed; attention/ vigilance; working memory; verbal learning; visual learning; reasoning and problem solving; social cognition assessed using social reasoning tasks for understanding and managing emotions taken from the Managing emotions subtest of the Mayer-Salovey-Caruso Emotional Intelligence Test (MSCEIT) which is a social reasoning test. The test comprises of vignettes of various situations, and options for coping with the emotions depicted in these vignettes [80, 81]. Participants are required to indicate the effectiveness of each solution ranging from one (very ineffective) to five (very effective). In validation studies, and in antipsychotic trials of stable patients, the MATRICS demonstrated excellent reliability, minimal practice effects and significant correlations with measures of functional capacity. It is hypothesised that there will be a group by time interaction (CRT vs TAU) on the total score of the MATRICS battery at the end of treatment approximately 6 months and at 8 months follow up.

### Secondary outcome measures

#### Change in specific cognitive domains

The MATRICS battery will also be used to assess change in specific cognitive domains for study participants. The processing speed, attention/ vigilance, working memory, visual learning, verbal learning, reasoning/ problem solving and social cognitive domains of the MATRICS battery will all be used as secondary outcome measures. It is hypothesised that there will be a group (CRT vs TAU) by time interaction on the MATRICS domain scores at the end of treatment and eight month follow up [61]

### Social cognitive measures

Changes in social cognition will be assed using The Reading the Mind in the Eyes Test [62], the Faux Pas Recognition Test [63] and the Managing Emotions subtest of the Mayer-Salovey-Caruso Emotional Intelligence Test (MSCEIT). It is hoped that each of these tests will tap into different components of the emotional processing stream [40]. The Reading the Eyes of the Mind Test will measure emotional perception as well as theory of mind, the Faux Pas Recognition Test will measure the participants awareness of emotional context or social sensitivity, and finally the MSCEIT is a measure of social as well as emotional reasoning.

### Real world functioning

Secondary outcome measures will include the Social and Occupational Functioning Assessment Scale (SOFAS) [64]. The SOFAS is a continuous scale (0–100) with verbal tethers so that higher scores represent superior functioning. It is similar to the Global Assessment of Functioning Scale however it does not include the severity of psychiatric symptoms. Again it is hypothesised that there will be a group (CRT vs TAU) by time interaction at the end of treatment and eight month follow up.

### Psychiatric symptoms

Secondary outcome measures will also include the scores on the disorganised and negative symptoms scales from the five factor Positive and Negative Syndrome Scale (PANSS) and the total score from the Clinical Assessment Interview for Negative Symptoms (CAINS) [67, 68]. The PANSS contains 30 items measuring psychopathology associated with schizophrenia, 7 items assess positive symptoms, seven items assessing negative symptoms and 16 items assess general psychopathology. A five factor model of the PANSS will be used to evaluate outcomes because CRT is thought to have a specific impact on negative and disorganised symptoms [82]. The CAINS is a 13 item interview for measuring negative symptoms associated with schizophrenia. It contains 9 items for assessing problems with motivation and pleasure and 4 items for assessing problems with emotional expression. Again it is hypothesised that there will be a group (CRT vs TAU) by time interaction at the end of treatment and eight month follow up.

### Violence risk

Violence risk will be assessed with the Historical and Clinical Risk Management Scale 20 (HCR-20) a measure of violence risk, sometimes referred to as violence proneness [51]. The HCR-20 is among the most widely used violence risk assessment schemes. The HCR-20 contains ten historical or static items, five current or clinical items and five future risk items. Both the clinical and risk items are thought to be dynamic in nature in that they can change over time and are amenable to therapeutic intervention. Because the historical items are static in nature only the dynamic items will be used as a secondary outcome measurement. Violence risk will only be measured at baseline and eight month follow up. Again it is hypothesised that there will be a group (CRT vs TAU) by time interaction. The HCR-20 will be rated approximately every six months by the treating multi-disciplinary team and researchers.

### Programme completion and recovery

It is hoped that there will be differences between participants receiving CRT compared to TAU on their ability to benefit from additional psychosocial treatment programmes offered. Participant ability to benefit from additional psychosocial treatment will be assessed with the Dundrum-3 Programme Completion scale rated by psychiatric registrars and by patients’ multidisciplinary teams blind to the intervention [78]. The Dundrum-3 is a structured clinical judgment instrument which assesses whether patients have participated in, engaged and benefited from psychosocial programmes and consists of seven items: physical health, mental health, drugs and alcohol, problem behaviours, self-care and activities of daily living, education occupation and creativity and family and social networks. Each item is rated on a five point scale with lower scores representing a higher level of participation and engagement. Patient ability to recover within a forensic setting will be assessed with the Dundrum-4 Recovery Scales [78]. The Dundrum 4 contains six items: stability, insight, therapeutic rapport, leave, dynamic risk and victim sensitivity. Similar to the DUNDRUM-3 Programme Completion Scale, each item of the DUNDRUM-3 is also rated on a five point scale with lower scores representing greater progress towards recovery. The Dundrum-3 Programme Completion Scale has been shown to significantly distinguish between levels of security within a forensic setting and the Dundrum-4 Recovery Scale has been shown to distinguish those given unaccompanied leave outside of a secure forensic setting. Programme completion and recovery will only be measured at baseline and eight month follow up. Again it is hypothesised that there will be a group (CRT vs TAU) by time interaction.

### Patient satisfaction measure

An interview developed by service users will be used to explore patient satisfaction with cognitive remediation [45]. The interview will inquire about the following areas: 1) task difficulty, 2) experience of sessions, 3) relationship with therapist and 4) impact of CRT on daily life. The interview will be administered by a social worker who will be independent of treatment and assessment teams, to the intervention and to other assessments. Patients will be reassured that their responses will be anonymously recorded i.e. that their names will not be connected with the feedback they provide.

### Proposed analysis

Data analysis will be carried out using an intention to treat methodology [83]. Data from all enrolled participants will therefore be used in the analysis regardless of their level of participation in the study. Missing data will be estimated using multiple imputation [84]

The interaction between time i.e. baseline vs. 6 month and treatment condition (CRT vs TAU) on our primary outcome variable (MCCB composite cognition score) will be examined rather than between group differences, because tests of group difference average Time 1 (baseline) and Time 2 (6 month) assessments across the groups and consequently will be less sensitive to change. The interaction between time and treatment condition will be assessed using a repeated measure ANOVA with age and gender entered as covariates. This analysis will also be repeated at follow up using all three data points.

An a priori estimate of statistical power was completed using G*Power 3.1 [85]. Assuming a correlation greater than or equal to .5 between baseline and 6 month MCCB composite and a medium effect size (i.e. f = .25), the power to detect a statistically significant interaction between time and treatment conditions (i.e. CRT vs TAU) is adequately powered i.e. greater than or equal to .80. Should we find a statistically significant time X treatment condition interaction, post-hoc probing of the interaction will be completed with Bonferroni corrections applied where appropriate to maintain an alpha of 0.05.

SPSS PROCESS Macro Model 4 will be used to explore mechanisms of action should we find a positive impact of CRT. For example, whether change in cognition leads to a change in functioning, or whether a change in negative symptoms leads to a change in functioning etc. Change score will be calculated by subtracting the scores at baseline from the sores following the intervention. Age and gender will be entered as covariates in all mediation analysis. Bootstrapping will be used to estimate indirect effects, and 95 % bias-corrected confidence intervals using 1,000 bootstrap samples will be applied. A confidence interval that does not contain zero indicates statistically significant mediation (*p* < 0.05).

## Discussion

Forensic mental health services have a dual role in treating and caring for patients with mental disorders while also protecting the public from recidivist acts of violence. This study aims to address both of these goals by attempting to alleviate or ameliorate the likely common underlying deficits leading to functional impairment and violence.

Recently there has been a shift in emphasis within the field of schizophrenia research from focusing on positive symptoms such as delusions and hallucinations to patients’ cognitive abilities and functional outcomes [86]. Positive symptoms can be fairly successfully treated with medication however to date there is a lack of effective pharmacological treatments for cognitive difficulties and negative symptoms [13, 14]. It is these difficulties which are associated with patients’ ability to function day to day [14]. Also many risk factors for violence for mentally disordered patients concern suboptimal functioning.

CRT appears to be an effective intervention for community patients with schizophrenia for improving cognitive deficits [42]. There is also evidence that the cognitive improvements brought about by CRT lead to improvements in patient functioning. Because forensic mental health patients tend to be hospitalised for a longer duration than those in the community there is an unrealised opportunity to improve cognition and restore functioning within forensic services [8].

The results of the proposed study will help to answer the question whether cognitive remediation therapy is an effective intervention strategy for forensic mental health patients. Specifically it will test whether a nationally representative cohort of forensic mental health patients with schizophrenia or schizoaffective disorder benefit from cognitive remediation and whether patients are satisfied with the intervention.

A focus on cognition as a primary treatment target also has the potential to reduce violence risk in two ways. First it could help patients who are cognitively impaired benefit from specialised psychosocial programmes targeting the risk of violence. Second it could improve general functional ability. By placing the emphasis on cognition and functional ability over symptoms any conflict between the two roles played by forensic mental health services could be reduced thus improving recovery and decreasing patients’ length of stay.

### Limitations

The protocol has some limitations. A weakness of the study is the lack of an active control group beyond treatment as usual (TAU). An additional weakness is that it will not be possible to keep medication constant for the duration of the study. The confined environment of a forensic hospital may also present fewer opportunities for practicing and applying cognitive skills. In a non-quantitative narrative review of over 100 psychological intervention studies it was estimated that extra-therapeutic factors such as the persons’ social environment accounted for approximately 40 % of the variance of the outcome of interventions [87]. Forensic services almost by definition limit patients’ freedom. And there can be conflict within forensic mental health services between safety and security on the one hand, and the provision of a therapeutic environment on the other. However these disadvantages will be offset by the consistency of the daily milieu for the intervention and TAU groups, a consistency that cannot be achieved for groups living in the community.

### Strengths

A major strength of the study is that CRT is being offered to a nationally representative cohort of forensic mental health patients with schizophrenia or schizoaffective disorder. The findings regarding efficacy and patient satisfaction will therefore inform whether CRT could or should be rolled out in forensic mental health services across other jurisdictions. A second strength of the study is the large battery of outcome measures for assessing the efficacy of the intervention, evaluating domains of cognition, functioning, symptoms, programme completion, recovery and violence risk. Finally the patients themselves will also play a role in assessing the usefulness of the intervention by participating in a confidential interview.

## Trial status

The trail is currently enrolling by invitation. Trial Registration: ClinicalTrials.gov Identifier: NCT02360813. First received: February 4, 2015.

## Abbreviations

CRT: cognitive remediation therapy
HCR-20: Historical-Clinical-Risk Management-20
TOPF: test of premorbid functioning
MATRICS: measurement and treatment research to improve cognition in schizophrenia
MCCB: MATRICS consensus cognitive battery
MSCEIT: Mayer-Salovey-Caruso emotional intelligence test
SOFAS: the social and occupational functioning assessment scale
PANSS: positive and negative syndrome scale
CAINS: clinical assessment interview for negative symptoms
MST: multi-systemic treatment
NFMHS: national forensic mental health service
CMH: Central Mental Hospital
CPZeq: chlorpromazine equivalents
SPSS-22: statistical package for the social sciences, version 22

## References

[1] Kennedy H (2002). Therapeutic uses of security: mapping forensic mental health services by stratified risk. Advances in Psychiatric Treatment.

[2] McFadyen JA (1999). Safe, sound and supportive: forensic mental health services. Br J Nurs.

[3] McInerney C, Davoren M, Flynn G, Mullins D, Fitzpatrick M, Caddow M (2013). Implementing a court diversion and liaison scheme in a remand prison by systematic screening of new receptions: a 6 year participatory action research study of 20,084 consecutive male remands. International Journal of Mental Health Systems.

[4] Packer IK (2009). Evaluation of criminal responsibility.

[5] Ireland. Criminal Law (insanity) Act 2010. Irish Statute Book. 2010 http://www.irishstatutebook.ie/2010/en/act/pub/0040/index.html.

[6] Buchanan A, Grounds A (2011). Forensic psychiatry and public protection. Br J Psychiatry.

[7] Andreasson H, Nyman M, Krona H, Meyer L, Anckarsäter H, Nilsson T (2014). Predictors of length of stay in forensic psychiatry: the influence of perceived risk of violence. Int J Law Psychiatry.

[8] Sharma A, Dunn W, O'Toole C, Kennedy HG (2015). The virtual institution: cross-sectional length of stay in general adult and forensic psychiatry beds. Int J Ment Health Syst.

[9] Fazel S, Fimińska Z, Cocks C, Coid J (2016). Patient outcomes following discharge from secure psychiatric hospitals: systematic review and meta-analysis. Br J Psychiatry..

[10] Davoren M, Byrne O, O'Connell P, O'Neill H, O'Reilly K, Kennedy HG (2015). Factors affecting length of stay in forensic hospital setting: need for therapeutic security and course of admission. BMC Psychiatry.

[11] Ricketts D, Carnell H, Davies S, Kaul A, Duggan C (2001). First admissions to a regional secure unit over a 16-year period: Changes in demographic and service characteristics. Journal of Forensic Psychiatry.

[12] Shah A, Waldron G, Boast N, Coid JW, Ullrich S (2011). Factors associated with length of admission at a medium secure forensic psychiatric unit. Journal of Forensic Psychiatry and Psychology.

[13] Leucht S, Tardy M, Komossa K, Heres S, Kissling W, Davis JM (2012). Maintenance treatment with antipsychotic drugs for schizophrenia. Cochrane Database Syst Rev.

[14] Keefe RS, Bilder RM, Davis SM, Harvey PD, Palmer BW, Gold JM (2007). Neurocognitive effects of antipsychotic medications in patients with chronic schizophrenia in the CATIE Trial. CATIE Investigators; Neurocognitive Working Group. Arch Gen Psychiatry.

[15] Kane JM, Correll CU (2010). Past and present progress in the pharmacologic treatment of schizophrenia. J Clin Psychiatry.

[16] Swartz MS, Perkins DO, Stroup TS, Davis SM, Capuano G, Rosenheck RA (2007). Effects of antipsychotic medications on psychosocial functioning in patients with chronic schizophrenia: findings from the NIMH CATIE study. Am J Psychiatry.

[17] Kirkpatrick B, Fischer B (2006). Subdomains within the negative symptoms of schizophrenia: commentary. Schizophr Bull.

[18] Green MF (1996). What are the functional consequences of neurocognitive deficits in schizophrenia?. American Journal of Psychiatry.

[19] Green MF, Kern RS, Braff DL, Mintz J (2000). Neurocognitive deficits and functional outcome in schizophrenia: are we measuring the "right stuff"?. Schizophr Bull.

[20] Green MF, Kern RS, Heaton RK (2004). Longitudinal studies of cognition and functional outcome in schizophrenia: implications for MATRICS. Schizophr Res.

[21] Velligan DI, Mahurin RK, Diamond PL, Hazleton BC, Eckert SL, Miller AL (1997). The functional significance of symptomatology and cognitive function in schizophrenia. Schizophr Res.

[22] Norman RM, Malla AK, McLean T, Voruganti LP, Cortese L, McIntosh E (2000). The relationship of symptoms and level of functioning in schizophrenia to general wellbeing and the Quality of Life Scale. Acta Psychiatr Scand.

[23] Lysaker PH, Davis LW (2004). Social function in schizophrenia and schizoaffective disorder: associations with personality, symptoms and neurocognition. Health Qual Life Outcomes.

[24] Kurtz MM, Moberg PJ, Ragland JD, Gur RC, Gur RE (2005). Symptoms versus neurocognitive test performance as predictors of psychosocial status in schizophrenia: a 1- and 4-year prospective study. Schizophr Bull.

[25] Milev P, Ho BC, Arndt S, Andreasen NC (2005). Predictive values of neurocognition and negative symptoms on functional outcome in schizophrenia: a longitudinal first-episode study with 7-year follow-up. Am J Psychiatry.

[26] Cohen AS, Saperstein AM, Gold JM, Kirkpatrick B, Carpenter WT, Buchanan RW (2007). Neuropsychology of the deficit syndrome: new data and meta-analysis of findings to date. Schizophr Bull..

[27] Kahn RS, Keefe RS (2013). Schizophrenia is a cognitive illness: time for a change in focus. JAMA Psychiatry.

[28] Brekke JS, Hoe M, Long J, Green MF (2007). How neurocognition and social cognition influence functional change during community-based psychosocial rehabilitation for individuals with schizophrenia. Schizophr Bull.

[29] Ventura J, Hellemann GS, Thames AD, Koellner V, Nuechterlein KH (2009). Symptoms as mediators of the relationship between neurocognition and functional outcome in schizophrenia: a meta-analysis. Schizophr Res.

[30] O'Connell M, Farnworth L (2012). Occupational Therapy in Forensic Psychiatry: A Review of the Literature and a Call for a United and International Response. Journal of International Medical Research.

[31] Williams E, Ferrito M, Tapp T (2014). Cognitive-behavioural therapy for schizophrenia in a forensic mental health setting. Journal of Forensic Practice.

[32] Blackburn R (2004). What works with mentally disordered offenders. Psychology Crime & Law.

[33] Lindqvist P, Skipworth J (2000). Finding the evidence in forensic rehabilitation. Br J Psychiatry.

[34] Lindqvist P, Skipworth J (2000). Evidence based rehabilitation in forensic psychiatry. Br J Psychiatry.

[35] Skeem JL, Steadman HJ, Manchak SM (2015). **Applicability of the** Risk**-**Need**-**Responsivity **Model to Persons With Mental Illness Involved in the Criminal Justice System**. Psychiatr Serv.

[36] Cullen AE, Clarke AY, Kuipers E, Hodgins S, Dean K, Fahy T (2012). A multisite randomized trial of a cognitive skills program for male mentally disordered offenders: violence and antisocial behavior outcomes. J Consult Clin Psychol.

[37] Rees-Jones A, Gudjonsson G, Young S (2012). A multi-site controlled trial of a cognitive skills program for mentally disordered offenders. BMC Psychiatry.

[38] O’Reilly K, Donohoe G, Coyle C, O’Sullivan D, Rowe A, Losty M (2015). Prospective cohort study of the relationship between neuro-cognition, social cognition and violence in forensic patients with schizophrenia and schizoaffective disorder. BMC Psychiatry.

[39] Lezak MD, Howieson DB, Bigler ED, Tranel D (2012). Neuropsychological Assessment (5th ed).

[40] Ochsner KN (2008). The social-emotional processing stream: five core constructs and their translational potential for schizophrenia and beyond. Biol Psychiatry.

[41] Carr A (2009). What Works with Children, Adolescents and Adults? A Review of Research on the Effectiveness of Psychotherapy.

[42] Wykes T, Huddy V, Cellard C, McGurk SR, Czobor P (2011). A meta-analysis of cognitive remediation for schizophrenia: methodology and effect sizes. Am J Psychiatry.

[43] Wykes T, Spaulding WD (2011). Thinking about the future cognitive remediation therapy--what works and could we do better?. Schizophr Bull.

[44] Cella M, Reeder C, Wykes T (2015). Lessons learnt? The importance of metacognition and its implications for Cognitive Remediation in schizophrenia. Front Psychol.

[45] Rose D, Wykes T, Farrier D, Doran AM, Sporle T, Bogner D (2008). What Do Clients Think of Cognitive Remediation Therapy?: A Consumer-Led Investigation of Satisfaction and Side Effects. American Journal of Psychiatric Rehabilitation.

[46] Spaulding WD, Reed D, Sullivan M, Richardson C, Weiler M (1999). Effects of cognitive treatment in psychiatric rehabilitation. Schizophr Bull.

[47] Taylor R, Cella M, Csipke E, Heriot-Maitland C, Gibbs C, Wykes T (2015). Tackling Social Cognition in Schizophrenia: A Randomized Feasibility Trial. Behav Cogn Psychother.

[48] Ahmed AO, Hunter KM, Goodrum NM, Batten NJ, Birgenheir D, Hardison E (2015). A randomized study of cognitive remediation for forensic and mental health patients with schizophrenia. J Psychiatr Res.

[49] Ullrich S, Keers R, Coid JW (2014). Delusions, anger, and serious violence: new findings from the MacArthur Violence Risk Assessment Study. Schizophr Bull.

[50] Witt K, Van Dorn R, Fazel S (2013). Risk factors for violence in psychosis: systematic review and meta-regression analysis of 110 studies. PLoS One.

[51] Webster CD, Douglas KS, Eaves D, Hart SD (1997). HCR–20: assessing risk for violence.

[52] Schizophrenia Commission (2012). The Abandoned Illness: A Report by the Schizophrenia Commission.

[53] Fazel S, Wolf A, Pillas D, Lichtenstein P, Långström N (2014). Suicide, Fatal Injuries, and Other Causes of Premature Mortality in Patients With Traumatic Brain Injury A 41-Year Swedish Population Study. JAMA Psychiatry.

[54] Wykes T, Steel C, Everitt B, Tarrier N (2008). Cognitive behavior therapy for schizophrenia: effect sizes, clinical models, and methodological rigor. Schizophr Bull.

[55] First MB, Spitzer RL, Gibbon M, Williams JBW. Structured Clinical Interview for DSM-IV-TR Axis I Disorders, Research Version, Patient Edition. (SCID-I/P): Biometrics Research, New York State Psychiatric Institute, November 2002.

[56] Corp IBM (2013). IBM SPSS Statistics for Windows, Version 22.0.

[57] Woods SW (2003). Chlorpromazine equivalent doses for the newer atypical antipsychotics. Journal of Clinical Psychiatry.

[58] Haddad P, Lambert T, Lauriello J (2010). Antipsychotic Long-acting Injections.

[59] Taylor D, Paton C, Kapur S (2012). The Maudsley prescribing guidelines in psychiatry.

[60] Wechsler D. Test of Pre-morbid Functioning - UK Version (TOPF UK). Pearson Corporation 2011.

[61] Nuechterlein KH, Green MF, Kern RS, Baade LE, Barch DM, Cohen JD (2008). The MATRICS Consensus Cognitive Battery, part 1: test selection, reliability, and validity. Am J Psychiatry.

[62] Baron-Cohen S, Wheelwright S, Hill J, Raste Y, Plumb I (2001). The "Reading the Mind in the Eyes" Test revised version: a study with normal adults, and adults with Asperger syndrome or high-functioning autism. J Child Psychol Psychiatry.

[63] Stone VE, Baron-Cohen S, Knight RT (1998). Frontal lobe contributions to theory of mind. J Cogn Neurosci.

[64] Rybarczyk B, Kreutzer J, DeLuca J, Caplan B (2011). Social and Occupational Functioning Assessment Scale (SOFAS). LXIII, Encyclopedia of Clinical Neuropsychology vol 1.

[65] Kay SR, Fiszbein A, Opler LA (1987). The positive and negative syndrome scale. (PANSS) for schizophrenia. Schizophr Bull.

[66] Kring AM, Gur RE, Blanchard JJ, Horan WP, Reise SP (2013). The Clinical Assessment Interview for Negative Symptoms (CAINS): final development and validation. Am J Psychiatry.

[67] Medalia A, Revheim N, Herlands (2009). Cognitive remediation for psychological disorders.

[68] Gastonguay LG, Beutler LE (2006). Principles of therapeutic change.

[69] Henggeler SW, Schoenwald KS, Borduin CM, Cunningham PB (1998). Multisystemic treatment of antisocial behaviour in children and adolescents.

[70] Davoren M, O'Dwyer S, Abidin Z, Naughton L, Gibbons O, Doyle E (2012). Prospective in-patient cohort study of moves between levels of therapeutic security: the DUNDRUM-1 triage security, DUNDRUM-3 programme completion and DUNDRUM-4 recovery scales and the HCR-20. BMC Psychiatry.

[71] Wampold B (2015). How important are the common factors in psychotherapy?. An update World Psychiatry.

[72] Huddy V, Reeder C, Kontis D, Wykes T, Stahl D (2012). The effect of working alliance on adherence and outcome in cognitive remediation therapy. J Nerv Ment Dis.

[73] Subramaniam K, Kounios J, Parrish TB, Jung-Beeman M (2009). A brain mechanism for facilitation of insight by positive affect. J Cogn Neurosci.

[74] Savine AC, Braver TS (2012). Local and global effects of motivation on cognitive control. Cogn Affect Behav Neurosci.

[75] Medalia A, Saperstein A (2011). The role of motivation for treatment success. Schizophr Bull.

[76] Kennedy HG, Bloom H, Webster CD (2007). Therapeutic Uses of Security: mapping forensic mental health services by stratifying risk. Essential Writings in Violence Risk Assessment and Management.

[77] Pillay SM, Oliver B, Butler L, Kennedy HG (2008). Risk stratification and the care pathway. Irish Journal of Psychological Medicine.

[78] Kennedy HG, O’Neill C, Flynn G, Gill P (2010). The DUNDRUM toolkit. Dangerousness understanding, recovery and urgency manual (the DUNDRUM Quartet) V1.0.21 (18/03/10), Four structured professional judgment instruments for admission triage, urgency, treatment completion and recovery assessments.

[79] Vinogradov S, Fisher M, Warm H, Holland C, Kirshner MA, Pollock BG (2009). The cognitive cost of anticholinergic burden: decreased response to cognitive training in schizophrenia. Am J Psychiatry.

[80] Mayer JD, Salovey P, Caruso DR (2002). Mayer-Salovey-Caruso. Emotional Intelligence Test (MSCEIT): User’s Manual.

[81] Mayer JD, Salovey P, Caruso DR, Sitarenios G (2003). Measuring emotional intelligence with the MSCEIT V2.0.. Emotion.

[82] Cella M, Reeder C, Wykes T (2014). It is all in the factors: effects of cognitive remediation on symptom dimensions. Schizophr Res.

[83] Montori VM, Guyatt GH (2001). Intention-to-treat principle. CMAJ.

[84] Rubin DB (1987). Multiple Imputation for Nonresponse in Surveys.

[85] Faul F, Erdfelder E, Lang AG, Buchner A (2010). G*Power 3: a flexible statistical power analysis program for the social, behavioral, and biomedical sciences. Biometrics.

[86] Wunderink L, Nieboer RM, Wiersma D, Sytema S, Nienhuis FJ (2013). Recovery in remitted first-episode psychosis at 7 years of follow-up of an early dose reduction/discontinuation or maintenance treatment strategy: long-term follow-up of a 2-year randomized clinical trial. JAMA Psychiatry.

[87] Lambert MJ, Barley DE, Norcross JC (2002). **Research summary on the therapeutic relationship and psychotherapy outcome**. *Psychotherapy relationships that work: Therapist contributions and responsiveness to patients* (pp. 17–).

